# Female Genital Mutilation/Cutting: Innovative Training Approach for Nurse-Midwives in High Prevalent Settings

**DOI:** 10.1155/2018/5043512

**Published:** 2018-03-15

**Authors:** Samuel Kimani, Tammary Esho, Violet Kimani, Samuel Muniu, Jane Kamau, Christine Kigondu, Joseph Karanja, Jaldesa Guyo

**Affiliations:** ^1^Africa Coordinating Centre for Abandonment of FGM/C, Kenyatta National Hospital, University of Nairobi, P.O. Box 19676-00202, Nairobi, Kenya; ^2^School of Nursing Sciences, Kenyatta National Hospital, P.O. Box 19676-00202, Nairobi, Kenya; ^3^Technical University of Kenya, P.O. Box 52426, Nairobi, Kenya; ^4^School of Public Health, Kenyatta National Hospital, P.O. Box 19676-00202, Nairobi, Kenya; ^5^Aga Khan University Hospital, 3rd Parklands Avenue, Limuru Rd., Nairobi, Kenya; ^6^Department of Clinical Chemistry, Kenyatta National Hospital, University of Nairobi, P.O. Box 19676-00202, Nairobi, Kenya; ^7^Department of Obstetrics and Gynaecology, Kenyatta National Hospital, University of Nairobi, P.O. Box 19676-00202, Nairobi, Kenya

## Abstract

**Background:**

Female genital mutilation/cutting (FGM/C) has no medical benefits and is associated with serious health complications. FGM/C including medicalization is illegal in Kenya. Capacity building for nurse-midwives to manage and prevent FGM/C is therefore critical.

**Objective:**

Determine the current FGM/C knowledge and effect of training among nurse-midwives using an electronic tool derived from a paper-based quiz on FGM/C among nurse-midwives.

**Methods:**

Nurse-midwives (*n*=26) were assessed pre- and post-FGM/C training using a quiz comprising 12 questions. The quiz assessed the following factors: definition, classification, determining factors, epidemiology, medicalization, prevention, health consequences, and nurse-midwives' roles in FGM/C prevention themes. The scores for individuals and all the questions were computed and compared using SPSS V22.

**Results:**

The mean scores for the quiz were 64.8%, improving to 96.2% *p* < 0.05 after training. Before the training, the following proportions of participants correctly answered questions demonstrating their knowledge of types of cutting (84.6%), link with health problems (96.2%), FGM/C-related complications (96.2%), communities that practice FGM/C (61.5%), medicalization (43.6%), reinfibulation (46.2%), dissociation from religion (46.2%), and the law as it relates to FGM/C (46.2%). The participants demonstrated knowledge of FGM/C-related complications with the proportion of nurse-midwives correctly answering questions relating to physical impact (69.2%), psychological impact (69.2%), sexual impact (57.7%), and social impact (38.5%). Additionally, participant awareness of NM roles in managing FGM/C included the following: knowledge of the nurse-midwife as counselor (69.2%), advocate (80.8%), leader (26.9%), role model (42.3%), and caregiver (34.6%). These scores improved significantly after training.

**Conclusion:**

Substantial FGM/C-related knowledge was demonstrated by nurse-midwives. They, however, showed challenges in preventing/rejecting medicalization of FGM/C, and there were knowledge gaps concerning sexual and social complications, as well as the specific roles of NM. This underscores the need to implement innovative FGM/C training interventions to empower health professionals to better respond to its management and prevention.

## 1. Introduction

Female genital mutilation/cutting (FGM/C) is a culturally entrenched persistent practice associated with socioeconomic repercussions on girls/women, families, and communities. The practice constitutes extreme form of violence, abuse, and violation of human rights against children, girls, and women [1, 2]. FGM/C entails all procedures involving partial or total removal of the female external genitalia or other injuries for cultural or nonmedical reasons [1, 3]. There are four defined types of FGM/C: namely, partial or total removal of the clitoris and/or the prepuce (clitoridectomy) (type I); partial or total removal of the clitoris and the labia minora, with or without excision of the labia majora (excision) (type II); narrowing of the vaginal orifice with creation of a covering seal by cutting and appositioning the labia minora and/or the labia majora, with or without excision of the clitoris (infibulation) (type III); and all other harmful procedures on the female genitalia for nonmedical purposes such as pricking, piercing, incising, scraping, and cauterization (type IV) [1].

FGM/C is practiced in 30 African countries (East, Northeast, and West Africa), the Middle East, Asia, Latin America, and in some Western nations among the migrant populations [1, 4–6]. In the Western nations, it is carried out during return home visiting trips among the diaspora community [7, 8], while some cutting has been performed by visiting/resident practitioners in countries where FGM/C is not traditionally practiced (Europe, North America, and Australia) [7, 9–12]. Globally, there are 200 million women/girls who have been forcibly cut, while 3.6 million risk being subjected to the practice each year [13, 14]. In Kenya, FGM/C is practiced by most communities, with exception of five (Luo, Luhya, Pokomo, Teso, and Turkana). The prevalence of FGM/C among 15- to 49-year-olds has consistently decreased from 37.6% (1998) to 32.2% (2003) and then to 27.1% (2008-2009), and currently, it is 21% [15]. The prevalence and support for FGM/C has been declining in both high prevalent and Western countries [16, 17]. However, the decline is disproportionately slower in high prevalent countries relative to the Western nations [17–20]. Thus, strategies should be devised and escalated to prevent new cuttings as well as mitigate the suffering of already cut girls/women. This calls for a scaled-up interventional program involving the health sector players in the abandonment strategy.

FGM/C is mainly performed by designated traditional practitioners or traditional birth attendants [21, 22]. However, recently, it has been reportedly performed by health care professionals (medicalization) in hospitals, clinics, homes, or neutral places using surgical tools, anesthetics, and antiseptics in the hope of mitigating the immediate complications associated with the cutting [13, 22, 23]. Medicalization also involves reinfibulation (reclosure) of deinfibulated external genitalia on women with FGM type III following delivery and related gynecologic procedures by doctors or nurse-midwives (NM) [21, 22, 24]. Medicalization has been supported and perpetuated mainly through supposedly justification, namely, harm reduction from immediate complications, fulfillment of cultural requirement, and respect for women cultural rights among many other reasons. Medicalized FGM/C has also been performed on the premise of Muslim religious requirements whose evidence is alleged to be captured in the hadith (recorded sayings and practices of the Prophet Mohamed) [25]. Additionally, FGM/C is financially lucrative, with health-care providers making economic gains, where the practice is legally outlawed [26–28]. Thus, anti-FGM/C strategies targeting and involving health professionals and the health system could have a significant impact toward its abandonment.

Notable FGM/C-related complications include physical, psychological, social, and sexual harms on women and by extension men [29, 30]. These deleterious effects span from immediate, short, and long term with some persisting for life [1, 31–34]. The impacts portend that health sector is strategically positioned for the frontline FGM/C-related abandonment interventions vide multidisciplinary role in management and prevention [35].

The health sector response to the FGM/C-related challenges could be through diverse roles and responsibilities played by various health professionals among them NM working in various service points. NM are professionals trained in both Nursing and Midwifery, work in health service points interfacing with women subjected to or at risk of FGM/C while seeking prenatal, childbirth, and postnatal services. These professionals form the bulk of health workforce, are extensively distributed, and provide primary as well as people-centered care such as reproductive health including in the remote and hard-to-reach areas [36]. Thus, NM should assume and play critical roles in management and preventive FGM/C-related interventions for the sake of girls and women [17, 37–39]. These roles can be accomplished in both health facility and community setting to accelerate abandonment of FGM/C. The promising high impact roles on the campaign against cutting include raising awareness on FGM/C-related health impacts [8], advocacy, counseling, managing, and caring roles [40]. These roles increase the status and thus ascribe significant respect for NM in the community. Therefore, performance and participation of NM in cutting of girls signals endorsement, perpetuation, and legitimization of FGM/C [22]. Thus, training the NM on key components of FGM/C would build their capacity to manage the FGM/C sequelae and prevent new cutting, while empowering women and advocating for the prevention of FGM/C and ending medicalization of the practice [41].

Despite significant investments and efforts to accelerate FGM/C abandonment, poor or lack of training for NM on competencies relevant to management of women/girls with cutting-related health complications has been reported [42]. This shortcoming persists despite WHO prioritizing training, development, review and update of FGM/C management guidelines [43], and establishment of curriculum for NM [44–46]. This depicts inadequate capacity for NM to manage and prevent FGM/C [35]. To address the gap, requisite knowledge, competencies, and skills on FGM/C should be integrated into pre- and in-service trainings. The training can involve innovative approaches, for example, the use of online e-tool to circumvent the bottlenecks of service interruptions associated with face-to-face sessions. Thus, establishing the baseline and training-related acquired knowledge on FGM/C following the use of innovative e-tool training is critical before rolling it out. We sought to determine the baseline and training-related knowledge using electronic tool (e-tool) derived from a paper-based quiz on FGM/C among NM in the practice prevalent counties in Kenya.

## 2. Materials and Methods

An electronic-derived paper-based pre- and posttraining quiz was used to assess knowledge on FGM/C themes among NM (*n*=26) from five prevalent counties in Kenya. The 12-question quiz was developed from UNFPA training modules on FGM/C for midwives [40]. The pretraining quiz was administered and completed in ten minutes before a 3-day training program. Similarly, the test was administered after the training inform of posttest quiz for the same timeline.

The quiz was structured into four parts, namely, demographic characteristics, instructions on how to answer, seven true-false, and five multiple-choice questions (MCQs). The demographic characteristics comprised the participant's age, professional qualifications, designation, and work station. The true-false questions delved on the types of FGM/C, relationship between cutting, maternal-infant morbidity and mortality, practicing communities, factors for cutting, and categories of cutters, respectively. Similarly, the MCQ questions captured information on medicalization including reinfibulation, preventive strategies including dissociating FGM/C from religion, practice-related complications, and the role of NM in managing and preventing the cutting.

The scores on the pre- and posttraining quizzes were determined by allocating one mark for each correct true-false question. Similarly, each response for the MCQs was allocated a mark each, resulting to five marks per question. The individual question and total overall (sum of the marks) scores across all the questions were computed in percentages using excel computer application. The scores in percentage were further categorized into >90 (excellent), 80–89 (very high), 70–79 (high), 60–69 (moderate), 50–59 (fair), 40–49 (poor), and <40 (very poor) performance levels. Thereafter, bar graphs were constructed with individual participant marks for pre- and posttraining. The participants' results were displayed using LCD projector for them to see, appreciate, and seek clarification on their performance. The pre- and posttest quiz data were entered in SPSS Version 22 per the question, cleaned, and analyzed. Descriptive statistics were generated and reported on, while a paired *t*-test was used to establish the statistical differences. The outputs generated were reported and discussed accordingly. Ethical clearance was obtained from the Kenyatta National Hospital/University of Nairobi Ethics and Research Committee (approval no. P572/09/2014).

The training was conducted using content on FGM/C extracted from the UNFPA training modules e-tool for midwives for 3 days. Training sessions using interactive, trainer-guided questions and group discussions through moderated plenaries were adopted. The session facilitators included researchers of diverse background relevant to FGM/C, notably Gynecologic/Obstetric, Nursing/Midwifery, Social Sciences, and Sexual Medicine affiliated to Africa Coordinating Centre for Abandonment of FGM/C (ACCAF).

The tool had an inbuilt electronic-supported quiz programmed in such a way that one must answer correctly the preceding questions before proceeding to the next. The tool enabled participants to retake questions until the correct response was achieved. Thus, all questions had to be answered correctly for one to successfully complete the training. The completion of the quiz allowed the participant to undertake a pledge. The pledge involved participants making a vow that they would not perform FGM/C and focus their efforts on advocating for its prevention in the community and with other health-care providers. In addition, the pledge detailed the roles NM should assume while managing and preventing FGM/C. Taking the oath committed NM to assume critical roles, for example, advocacy, counseling, and role modelling in campaigns to accelerate the abandonment of FGM/C.

## 3. Results

### 3.1. Demographic Characteristics of the Participants

A total of 26 participants completed the baseline and posttraining quizzes. Most (80.8%) of them were females aged 40 ± 6.38 (mean ± SD) (range 27–53) years (Table 1). Their qualifications in Nursing/Midwifery included diploma (73.1%), Bachelor's degree (11.5%), Masters (7.7%), and Certificate (7.7%), respectively. Similarly, NM held different designations in Nursing/Midwifery, namely, Nursing/Midwifery Officer I (N/MOI) (80.8%), Senior Nursing Officer (SNO) (7.7%), Senior Certificate Midwife/Nurse (SCM/N) (7.7%), Enrolled Community Nurse/Midwife (ECN/M) (3.8%), and Program Officer (PO) (3.8%). The NM worked in health facilities across five counties of Kenya as follows: Narok (26.9%), Elgeyo-Marakwet (19.2%), Baringo (19.2%), Samburu (15.4%), West Pokot (15.4%), and the Nairobi County (3.8%).

### 3.2. Cumulative Scores of Knowledge on FGM/C-Related Contents during the Two Phases

**Figure 1:**
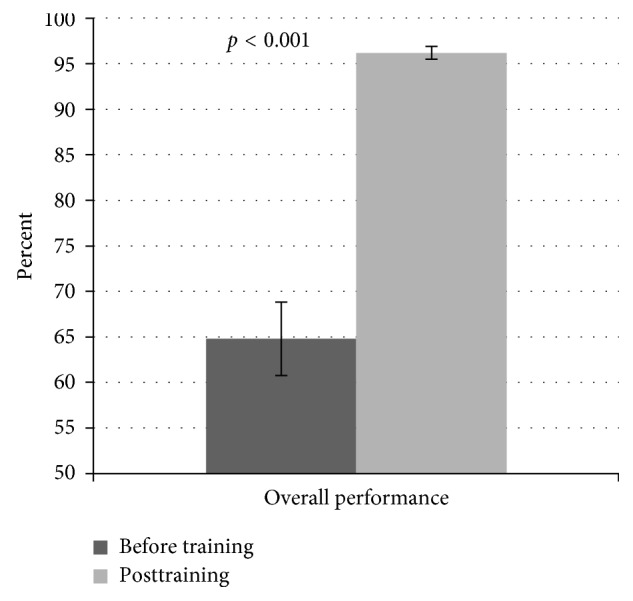
Cumulative scores of knowledge on FGM/C-related contents during the two phases. The knowledge score on all FGM/C-related components was 64.8% with significant improvement (96.2%) (*t*(25) = 7.408, *p* < 0.001) upon training.

The mean score on FGM/C-related contents across the 12 questions undertaken during the baseline and posttraining is presented. The performance levels were moderate (64.8%) during baseline relative to excellent (96.2%) posttraining (Figure 1). Further analysis showed a significant (*t*(25) = 7.408, *p* < 0.001) improvement after the 3-day training.

### 3.3. Knowledge on Specific FGM/C-Related Themes during the Two Phases

**Figure 2:**
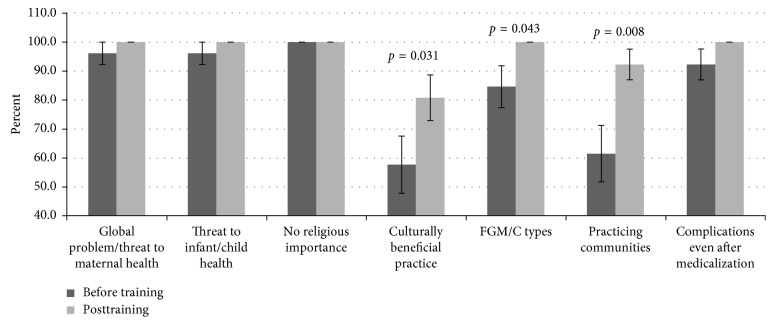
The difference between the specific FGM/C knowledge of participants before and after the training. The knowledge of FGM/C as a global health problem, threat to maternal-child-infant-health, and dissociating it from religion was high. However, NM supported the cultural benefits of FGM/C, while knowledge of communities who support the practice was average but significantly (*t*(25) = −2.287, *p*=0.031) improved on training.

The knowledge on the types of FGM/C was very high (84.6%) during the baseline with significant (*t*(25) = 2.132, *p*=0.043) improvement (100%) posttraining (Figure 2). Similarly, FGM/C as a global health problem and threat to maternal-child-infant-health was excellent (96.2% versus 100%) at baseline as well as during posttest.

Questions concerning communities for which cutting of girls is permissible and factors perpetuating the practice, scores were moderate (61.5%) at pretest while during posttest excellent scores (92.3%) were realized. The difference between the two were statistically significant (*t*(25) = −2.857, *p*=0.008). The assumption that FGM/C is culturally beneficial was supported by 57.7% of the participants during the pretest and (80.8%) on posttraining, with significant (*t*(25) = −2.287, *p*=0.031) improvement upon training. However, despite the improvement, questions regarding culture were incorrectly answered on the first go (>90%) compared to other components of FGM/C. The participants demonstrated excellent (100%) knowledge on the need to dissociate religion from FGM/C during both quizzes. Concerning association of FGM/C with health consequences, regardless of the practitioner (traditional excisors or health-care providers), excellent scores (96.2% versus 100%) were achieved during both tests. The difference between the two quizzes was, however, not statistically significant.

### 3.4. Knowledge on FGM/C-Related Facts Relevant to Resist/Prevent Medicalization

**Figure 3:**
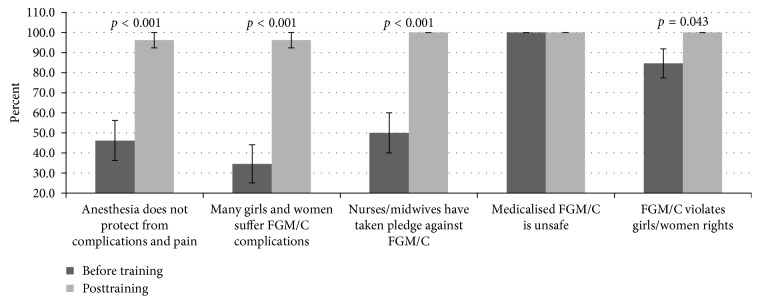
Participants' scores concerning issues relating to the medicalization of FGM/C. Before the training, few participants were aware of the existence of taking a pledge to prevent FGM/C. However, many participants were aware of FGM/C being a violation of women/girl rights and that medicalized cutting was unsafe. Furthermore, all the responses improved significantly (*p* < 0.001) upon training.

The capacity of participants to reject medicalization of FGM/C was assessed using a five-choice question. The response “*anesthesia will not protect the girl from long-term complications and pain*” had 46.2% concurring during baseline, with significant (t (25) = −4.372, *p* < 0.001) improvement (96.2%) after the training (Figure 3). Only 34.6% correctly responded to the statement “*No*, *I have seen many girls suffer FGM/C complications even after medicalization*” during baseline. However, after training, significant (*t*(25) = −5.494, *p* < 0.001) improvement (96.2%) was achieved. The response “*No, NM have taken a pledge never to perform FGM/C as it is not medically necessary and causes harm”* received 50% support during the baseline, with significant (*t*(25) = −5.000, *p* < 0.001) improvement (100%) during posttraining. Regarding the statement “*Yes, I will, It will be safer for your daughter if I perform it,*” the participants (100%) concurred to the inaccuracy of the choice during both quizzes. Additionally, the consensus to the response “*No, FGM/C violates women and girls human rights*” during baseline was very high (84.6%) and significantly (*t*(25) = −2.132, *p*=0.043) improved (100%) following the training.

### 3.5. Knowledge on Facts Dissociating Religion from FGM/C Relevant to Resist/Prevent Medicalization

**Figure 4:**
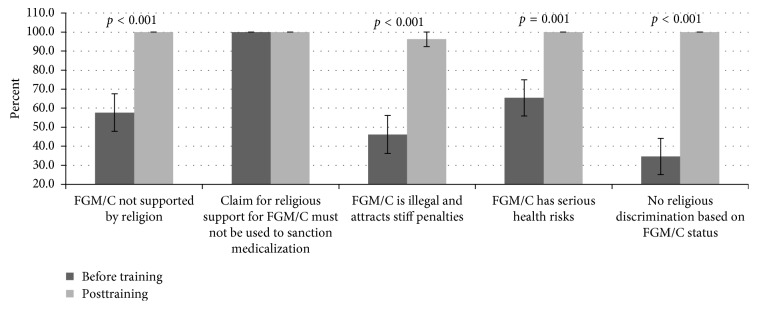
: Participants' view on the FGM/C in relation to religion. The knowledge on religious discrimination based on FGM/C status and its requirement by religion as well as its illegality were poorly scored during baseline with significant (*p* < 0.001) improvement after training.

The capacity of participants to reject the medicalization of FGM/C through religious clarification was assessed using a five-choice question. The choice was deemed correct when consistent with known knowledge/facts on the link between religion and cutting that could be used to object/resist offering medicalized FGM/C (Figure 4). Participants were presented with a scenario in which they were asked what information they should provide to a family who requested that their daughter be cut. Only 57.7% supported the response “*No, there is no evidence that FGM/C is required by any religion”* during the baseline, while training significantly (*t*(25) = −4.282, *p* < 0.001) improved (100%) the performance. There was a unanimous (100%) objection to the statement “*Yes, if it is your religion then you must and I will help you”* in both phases. On the choice “*No, FGM is illegal in the country and we would both face stiff penalties if I were to perform it,”* only 46.2% concurred with the response before training. The training significantly (*t*(25) = −5.000, *p* < 0.001) improved (96.2%) the response. Only 65.4% supported the response “*No, FGM/C has serious risks*” during before training, with significant (*t*(25) = −3.638, *p*=0.001) improvement (100%) in posttest. There was poor (34.6%) support on the response that “*No, there is no religious discrimination based on FGM/C status*” during the baseline. These scores significantly (*t*(25) = −6.872, *p* < 0.001) improved (100%) after the training.

### 3.6. Knowledge on FGM/C-Related Facts Relevant to Resist/Prevent Reinfibulation

**Figure 5:**
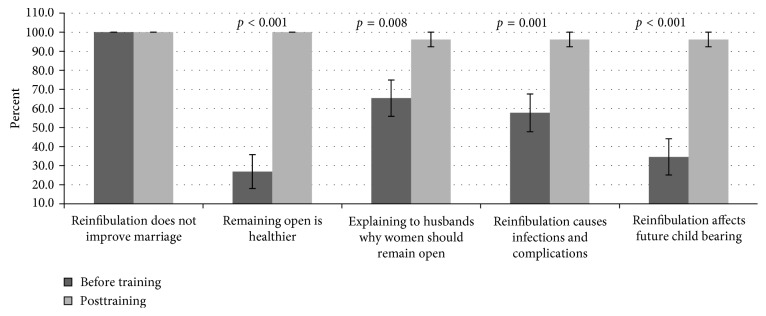
Participants' response regarding reinfibulation.

The capacity of participants to resist reinfibulation was assessed using a question with five choice answers (Figure 5). Participants were asked what response they would provide if a woman requested reinfibulation supposedly under pressure from her husband. There was unanimous (100%) objection to the response “*Yes, I will reinfibulate you because this will help improve your marriage”* during both quizzes. Only 26.9% concurred with the statement “*No, it is much healthier for you to remain open*” during baseline, and this significantly (*t*(25) = −8.238, *p* < 0.001) improved (100%) after the training. On responding to “*No, I can speak to your husband and explain why you should remain open if you would like,”* 65.6% supported during baseline, while training significantly (*t*(25) = −2.857, *p*=0.008) improved (96.2%) the performance. Additionally, the consensus on “*reinfibulation could cause infections and other complications”* was 57.7% during pretest with significant (*t*(25) = −3.953, *p*=0.001) improvement (96.2%) on posttest. Poor (34.6%) scores on the statement *“reinfibulation could affect future child bearing*” during baseline compared to significantly (*t*(25) = −6.325, *p* < 0.001) improved (96.2%) scores on posttest.

Participants' knowledge was poor to moderate on the importance of the need to leave the woman deinfibulated. However, the NM were willing to convince the husband on the need for the wife to remain deinfibulated and were unwilling to perform reinfibulation. The performance on the responses improved significantly after training.

### 3.7. Knowledge on FGM/C-Related Health Consequences

**Figure 6:**
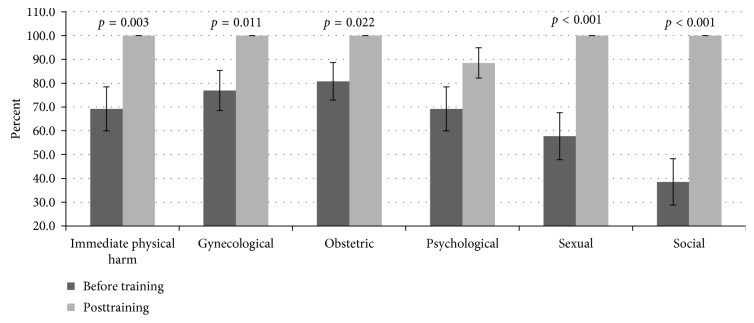
Participants were highly knowledgeable on gynecological (79.6%), obstetric (80.8%), immediate (69.2%), and psychological (69.2%) harms, while poor on sexual (57.7%) and social (38.5%) complications associated with FGM/C. Furthermore, the 3-day training significantly (*p* < 0.05) improved the scores on all the complications except the psychological consequences.

There was varied knowledge levels on health consequences associated with FGM/C including immediate physical (69.2%), gynecological (79.6%), obstetric (80.8%), psychological (69.2%), sexual (57.7%), and social (38.5%) harms, during baseline (Figure 6). The scores improved as follows: immediate physical (*p*=0.003), gynecological (*p*=0.011), obstetric (*p*=0.022), sexual (*p* < 0.001), and social harms (*p* < 0.001), during posttest. However, the change in scores on the FGM/C-related psychological complications was not statistically significant after the training.

### 3.8. Knowledge on the Roles Participants Should Assume in the Management and Prevention of FGM/C

**Figure 7:**
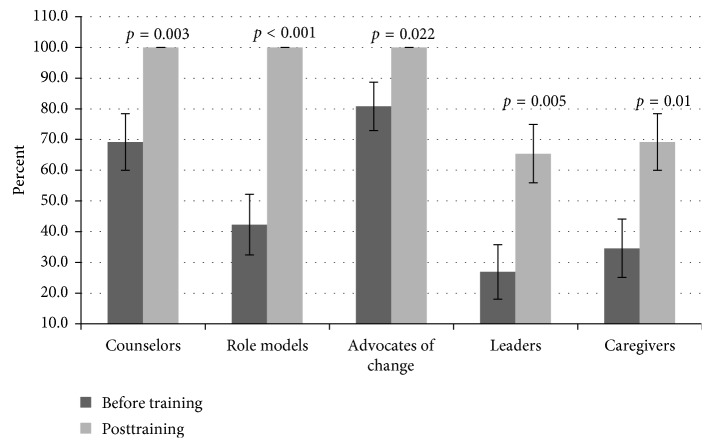
Knowledge on the roles participants should assume in the management and prevention of FGM/C. Participants scored high on advocacy (80.8%) and average on counselor's (69.2%) roles. However, poor knowledge on role models (42.3%), leaders (26.9%), and caregivers' (34.6%) roles were demonstrated during baseline. The scores improved significantly (*p* < 0.05) in all the components after training.

The participants demonstrated varied knowledge levels on the roles NM should assume in the management and prevention of FGM/C (Figure 7). The scores included counselor (69.2%), role model (42.3%), advocate of change (80.8%), leader (26.9%), and caregiver (34.6%) during the baseline assessment. Further analysis revealed that the scores in all the role categories improved significantly (*p* < 0.05) following the 3-day training.

## 4. Discussion

Our findings show that the (i) nurse-midwives were knowledgeable on the types of cutting and the FGM/C-related physical health complications as well as their roles in counseling and advocacy. However, (ii) their knowledge on communities that practice cutting of girls was inadequate and erroneously thought FGM/C is culturally beneficial, (iii) they were less knowledgeable on religious facts relevant in resisting/preventing medicalization including reinfibulation, (iv) they had challenges in identifying FGM/C-related sexual and social complications, (v) they had inadequate knowledge on their roles as caregiver, role model, and leader in management and prevention of FGM/C, and (vi) they improved their knowledge level on all the FGM/C-related themes after training.

The NM were professionally experienced, in their fourth decade, females and with diploma qualifications. These professionals are hired and retained in the public service, the main employer and provider of health services in Kenya. Their terms of employment are permanent and pensionable with the public sector, making it competitive with less staff turnover. The NM are hired immediately after qualifying from professional training colleges, particularly in the hard-to-reach counties where local human capacity supply does not match the demand [47]. The NM FGM/C-related knowledge is expected to be high, given their age and experience, and qualifications in Nursing/Midwifery, and their work stations in reproductive health service points and girls cutting prevalent counties. This assumption is on the premise that learning and acquisition of knowledge can be through daily encounter with problems, troubleshooting, and hands on experience. This mode of learning has been used to acquire competencies in health-related professions together with well-laid foundation of professional training.

The level of knowledge on FGM/C-related themes was moderate but improved substantially after training. The training process was effective, thanks to suitable tool, adequate content and delivery methods, and the relevance of FGM/C issue to the NM. Inadequate or lack of anchoring of FGM/C content in the training curricula, absence of refresher, and in-service capacity building interventions for NM could have contributed to poor pretraining scores [28]. These assumptions are consistent with findings from a study in Sudan, a high FGM/C prevalent country, which showed midwives training only involved a brief mention of female circumcision [28]. The aforesaid together with the proposal for educational tools and guidelines to support FGM/C training of health professionals could promote knowledge acquisition [48]. It is worth noting, however, that the environment for midwifery training and practice in Kenya and Sudan differ in many aspects.

The majority of Kenyan NM complete professional training aged 21–24 years. From the aforesaid, and going by their average age during this study, we estimate they could have been in training when global attention towards FGM/C had not gained momentum. FGM/C as a public health and a human rights problem began receiving significant global attention 3 decades ago [44, 46, 49–54]. However, tools and instruments for promoting health-care providers' capacity to address problems namely, declaration of training a priority strategy, FGM/C management guidelines, and curriculum for NM at global level spearheaded by WHO commenced in 2001 [44–46]. In Kenya, the first training and management guidelines for supporting health-care providers were developed and approved a decade after the WHO initiative [54]. Subsequently, the impetus to fight FGM/C was reinforced through the enactment of the prohibition of FGM act of 2011 [55].

Moreover, the knowledge levels on some specific FGM/C-related components, namely, the types of cutting, cutting-related physical complications, and its threat to maternal-child-infant-health, were highly consistent with recent findings from Gambia, a high FGM/C prevalent West African country [56]. The on-job learning as opposed to structured training was attributed to the high knowledge levels from health professionals from Gambia and Sudan [28, 57–59], while training imparted confidence and skills to offer care and counseling among women subjected to FGM in Somalia [60]. The health professionals frequently encounter mothers and girls with various types of FGM/C in their diverse health service points [1, 4–6], contributing the high scores. This supposition is consistent with documented evidence among Sudanese midwives who demonstrated high knowledge due to exposure to women with FGM/C-related issues [28, 59], another evidence of the effectiveness of on-job training. In addition, experience and knowledge are accrued from managing and caring for women with the FGM/C-related complications [28, 34, 61–64] and participation in meeting discussing vital health statistics.

Interestingly, cultural-related knowledge regarding FGM/C, namely, the cutting communities, and supposedly cultural beneficial effect of cutting was inadequate but improved on training. The improvement, however, was short of the 90% mark compared to other FGM/C themes. This indicates resistance/immunity of cultural issues to training and/or education, as well as the strong role of culture on practices and beliefs whether good or bad. This evidence is consistent with Gambian situation where health professionals supported the continuation of FGM/C, intended to subject their daughters to cut, while some had already performed medicalized FGM/C [65]. The health-care professionals belong and prescribe to cultural communities and thus are obliged to comply with societal cultural requirements to avoid sanctions and prohibitions [28]. Fear of social sanctions and cultural banishment strongly contribute to the persistence of the FGM/C, including medicalization [28]. Additionally, inadequate awareness of communities that practiced FGM/C by the providers points to lack of cultural competences probably due to inadequate community dialogues and interactions [57, 58, 61, 66] similar to nurses practicing in high-income countries attending to migrant communities [57, 58, 61, 66].

The knowledge of medicalized FGM/C and legal-related facts critical for rejecting/preventing cutting was inadequate but tremendously improved after training. This is despite NM being the frontline health professionals in the management and prevention of FGM/C at both health facility and community setting. The inconsistencies are corroborated by findings, indicating NM were under pressure to perform reinfibulation considered as normal and promoted by senior midwives [28, 60]. Elsewhere, midwives reported acknowledging FGM/C to be legally banned but did not fear the punishment [28]. Furthermore, the findings on lack of knowledge on reinfibulation among NM are due to lack of training on FGM/C during preservice and in-service. Infibulation is the most extreme form of FGM/C associated with serious health outcomes [34]. Therefore, it is imperative that NM be empowered to recognize, manage, and resist reinfibulation for the sake of the health of women and girls. However, our findings are consistent with those of midwives from UK whose reports indicated that few midwives had meaningful knowledge on the diagnosis and management of infibulation in labor and during birth in the UK [59]. The lack of capacity building on FGM/C-related legal status, updates, cascading, and enforcement of the law to the community and institutions may have played a big role.

Knowledgeability of FGM/C-related health impacts notably obstetric and gynecologic was high, while that of psychological, sexual, and social was poorly scored. The participants' work experience especially frequent encounter with physically related reproductive problems [57, 58] points to the attention they receive as opposed to neglect of the former [8, 34, 35]. Indeed, elsewhere nurses were less knowledgeable on FGM/C-related sexual impact [58, 59, 66]. This is consistent with evidence corroborated on the neglect to reproductive-related psychological problems, namely, puerperal psychosis and depression considered as indirect remote impacts in maternal health intervention [67–69]. FGM/C-related social effects, namely, early/forced/child marriage, family breakups (divorce and/or separation), prostitution, discrimination, and stigmatization were poorly scored [14, 35, 70]. These are emerging issues pertinent to FGM/C that are increasingly being noted but rarely mentioned in literature, thus calling for health-care professionals' empowerment to mitigate them consistent with similar suggestions [57, 61].

Finally, the NM were clear on their roles on counseling and advocacy while managing and preventing FGM/C, while traits like role modelling, leader, and caregiver roles were poorly understood. Similar findings have been documented [60, 71–74] among the midwives from Somali, as survivors of cutting as well as their accrued experience from caring for cut women and girls [60]. However, the variance with our findings is unexpected because NM reported responding to obstetric and gynecologic clients, thus expecting to score high on the caregivers role. The level of their knowledge could thus have been affected by lack of professional training and continuous professional development initiatives similar to other reports [57, 59, 60]. Similarly, the inadequacies have been attributed to the inability of the health system to allow NM to execute these roles [75, 76].

Therefore, improved overall knowledge on FGM/C themes demonstrates that our structured training is effective in imparting knowledge. Thus, the UNFPA training module is a promising innovative tool and potentially effective in delivering comprehensive knowledge and competencies on FGM/C. The tool could be used electronically in places where computers and smartphones are available as well as on a paper-based mode in group-teaching settings. This tool is appropriate in low-income settings with high prevalence of FGM/C, hard-to-reach communities, and the diaspora communities practicing FGM/C, where service disruption associated with face-to-face training mode may be a problem. There is need, however, to pilot the model to determine its applicability for teaching other health professionals. Thus, the tool has potential for skill and knowledge transfer while guaranteeing service delivery for the working professionals [40]. A practical approach to training should involve integrating FGM/C into the basic and postbasic NM curricula, continuing professional development programs, apprenticeship, and mentorship programs. FGM/C protocols and standard operating procedures for management and prevention should be developed and adopted in hospital and community settings. Additionally, a monitoring and evaluation mechanism for FGM/C-related interventions should be developed to oversee the implementation of the aforesaid issues. These strategies should be replicated to medical education and training of other frontline responders to FGM/C-related issues.

The notable weaknesses regarding this study include the small nonrandom sample. Despite the size, the representation of participants was from high FGM/C prevalent counties, reportedly with poor maternal-infant-child statistics. Thus, the generated evidence can serve to inform policy, practice, programming, and investments for FGM/C in those counties. Furthermore, the participants were qualified and experienced, whose inputs are critical in changing the practice. Finally, the evaluation on the knowledge acquisition was on short-term basis, and therefore, the need was to evaluate the scores after several months or years to establish retention.

In conclusion, substantial FGM/C-related knowledge is demonstrated by nurse-midwives. However, they are found with challenges in preventing/rejecting medicalization of FGM/C and have knowledge gaps concerning sexual and social complications, as well as their specific roles. This underscores the need to implement innovative FGM/C training interventions to empower health professionals to better respond to its management and prevention. This suggestion is in line with the UNFPA-funded activity of integrating FGM/C components into the university medical-related training curricula spearheaded by ACCAF. The tool will be instrumental in supporting, planning, and implementation of the curricula and roadmap for producing health professionals with requisite competencies to manage and prevent FGM/C in Kenya in line with recent recommendations for educational tools and guidelines for FGM/C training [48].

## Figures and Tables

**Table 1:** Demographic characteristics of the participants (*n*=26).

Characteristic	Number (*n*=26)	Percent
*Gender*		
Male	5	19.2
Female	21	80.8
*Professional qualifications*		
Masters	2	7.7
BScN	3	11.5
Diploma	19	73.1
Certificate	2	7.7
*Counties of work*		
Nairobi	1	3.8
Narok	7	26.9
Samburu	4	15.4
Baringo	5	19.2
West Pokot	4	15.4
Elgeyo-Marakwet	5	19.2
*Designation*		
N/MO1	21	80.8
SCM/N	1	3.8
SNO	2	7.7
ECN/M	1	3.8
Program Officer	1	3.8

## Abbreviations

ACCAF: Africa Coordination Centre for Abandonment of Female Genital Mutilation/Cutting
ECN/M: Enrolled Community Nurse/Midwife
FGM/C: Female genital cutting/mutilation
HCPs: Health care providers
HIV: Human immunodeficiency virus
NM: Nurse-Midwives
N/MOI: Nursing/Midwifery Officer I
PO: Program Officer
SCM/N: Senior Certificate Midwife/Nurse
SNO: Senior Nursing Officer
UNFPA: United Nations Population Fund
UK: United Kingdom
WHO: World Health Organization.

## References

[1] WHO (November 2017). Eliminating female genital mutilation: an interagency statement. http://www.unfpa.org/sites/default/files/pub-pdf/eliminating_fgm.pdf.

[2] Reza A., Mercy J. A., Krug E. (2001). Epidemiology of violent deaths in the world. *Injury Prevention*.

[3] Rymer J. (2003). Female genital mutilation. *Current Obstetrics and Gynaecology*.

[4] PRB (2013). *Ending Female Genital Mutilation/Cutting: Lessons from a Decade of Progress*.

[5] UNICEF (2014). *Female Genital Mutilation/Cutting: What Might the Future Hold?*.

[6] Yoder P. S., Wang S., Johansen E. (2013). Estimates of female genital mutilation/cutting in 27 African countries and Yemen. *Studies in Family Planning*.

[7] Elgaali M., Strevens H., Mårdh P.-A. (2005). Female genital mutilation—an exported medical hazard. *European Journal of Contraception and Reproductive Health Care*.

[8] Berg R. C., Denison E. M. (2013). A realist synthesis of controlled studies to determine the effectiveness of interventions to prevent genital cutting of girls. *Paediatrics and International Child Health*.

[9] Litorp H., Franck M., Almroth L. (2008). Female genital mutilation among antenatal care and contraceptive advice attendees in Sweden. *Acta Obstetricia et Gynecologica Scandinavica*.

[10] Thierfelder C., Tanner M., Bodiang C. M. K. (2005). Female genital mutilation in the context of migration: experience of African women with the Swiss health care system. *European Journal of Public Health*.

[11] Johnson-Agbakwu C. E., Helm T., Killawi A., Padela A. I. (2014). Perceptions of obstetrical interventions and female genital cutting: insights of men in a Somali refugee community. *Ethnicity and Health*.

[12] Moeed S. M., Grover S. R. (2012). Female genital mutilation/cutting (FGM/C): survey of RANZCOG fellows, diplomates & trainees and FGM/C prevention and education program workers in Australia and New Zealand. *Australian and New Zealand Journal of Obstetrics and Gynaecology*.

[13] UNICEF (2013). *Levels and Trends in Child Mortality Report*.

[14] UNICEF (September 2016). *Female Genital Mutilation/Cutting: A Global Concern*.

[15] Kenya National Bureau of Statistics (2014). *Kenya Demographic and Health Survey 2014*.

[16] Varol N., Fraser I. S., Ng C. H., Jaldesa G., Hall J. (2014). Female genital mutilation/cutting—towards abandonment of a harmful cultural practice. *Australian and New Zealand Journal of Obstetrics and Gynaecology*.

[17] UNFPA (2015). *Demographic Perspectives on Female Genital Mutilation*.

[18] European Parliament (June 2016). Combating violence against women. European Parliament resolution on the current situation in combating violence against women and any future action (2004/2220(INI)). http://www.europarl.europa.eu/sides/getDoc.do?pubRef%20=%20-//EP//TEXT+TA+P6-TA-2006-0038+0+=DOC+XML+V0//EN.

[19] Berg R. C., Denison E. (2012). Interventions to reduce the prevalence of female genital mutilation/cutting in African countries. *Campbell Systematic Reviews*.

[20] Leye E., Sabbe A. (2009). *Responding to Female Genital Mutilation in Europe. Striking the Right Balance Between Prosecution and Prevention*.

[21] Serour G. (2013). Medicalization of female genital mutilation/cutting. *African Journal of Urology*.

[22] WHO (2010). *Global Strategy to Stop Health-Care Providers from Performing Female Genital Mutilation*.

[23] Jaldesa G. W., Askew I., Njue C., Wanjiru M. (2005). *Female Genital Cutting among the Somali of Kenya and Management of its Complications*.

[24] Serour G. (2010). The issue of reinfibulation. *International Journal of Gynecology and Obstetrics*.

[25] Kaphle S. R. (2000). *Report of Qualitative Research on the Communication Channels in Use in Somalia*.

[26] Shell-Duncan B. (2001). The medicalization of female “circumcision”: harm reduction or promotion of a dangerous practice?. *Social Science and Medicine*.

[27] Njue C., Askew I. (2004). *Medicalisation of Female Genital Cutting among the Abagusii in Nyanza Province, Kenya, Frontiers in Reproductive Health*.

[28] Berggren V., Abdel S., Bergrstrom S., Johansson E., Edberg A. (2004). An explorative study of Sudanese midwives’ motives, perceptions and experiences of re-infibulation after birth. *Midwifery*.

[29] Vloeberghs E., van der Kwaak A., Knipscheer J., van den Muijsenbergh M. (2012). Coping and chronic psychosocial consequences of female genital mutilation in the Netherlands. *Ethnicity and Health*.

[30] Almroth L., Almroth-Berggren V., Hassanein O. M. (2001). Male complications of female genital mutilation. *Social Science and Medicine*.

[31] Kaplan A., Hechavarría S., Martín M., Bonhoure I. (2011). Health consequences of female genital mutilation/cutting in the Gambia, evidence into action. *Reproductive Health*.

[32] WHO (2000). *A Systematic Review of the Health Complications of Female Genital Mutilation Including Sequelae in Childbirth*.

[33] Morison L., Scherf C., Ekpo G. (2001). The long-term reproductive health consequences of female genital cutting in rural Gambia: a community-based survey. *Tropical Medicine and International Health*.

[34] WHO (2006). Female genital mutilation and obstetric outcome: WHO collaborative prospective study in six African countries. *The Lancet*.

[35] Kimani S., Muteshi J., Njue C. (2016). *A Synthesis of the Evidence: Health Impacts of FGM/C, FGM/C Research Consortium Programme Report*.

[36] WHO (June 2016). *WHO Nursing and Midwifery Progress Report 2008–2012*.

[37] ICM (July 2016). *International Definition of a Midwife*.

[38] Ball T. (2008). Female genital mutilation. *Nursing Standard*.

[39] UNDESA (2014). *World Urbanization Prospects: The 2014 Revision, Highlights (ST/ESA/SER.A/352)*.

[40] UNFPA (June 2016). *Engaging Midwives in the Global Campaign to End Female Genital Mutilation, Toolkit*.

[41] Population Council (June 2016). *Training Can Enhance Providers’ Management of FGM/C and Willingness to Advocate Against the Practice*.

[42] WHO (2011). *Female Genital Mutilation Programmes to Date: What Works and What Doesn’t*.

[43] WHO (2016). *WHO Guidelines on the Management of Health Complications from Female Genital Mutilation*.

[44] WHO (2001). *Management of Pregnancy, Child Birth and the Postpartum Period in the Presence of Female Genital Mutilation: Technical Consultation held in Geneva in 1997*.

[45] WHO (2001). *Female Genital Mutilation the Prevention and the Management of the Health Complications Policy Guidelines for Nurses and Midwives*.

[46] WHO (2001). *Female Genital Mutilation Integrating the Prevention and the Management of the Health Complications into the Curricula of Nursing and Midwifery—A Student’s Manual*.

[47] Gross M., Riley P., Kiriinya R. (2010). *The Impact of an Emergency Hiring Plan on the Shortage and Distribution of Nurses in Kenya: The Importance of Information Systems*.

[48] Zurynski Y., Sureshkumar P., Phu A., Elliott E. (2015). Female genital mutilation and cutting: a systematic literature review of health professionals’ knowledge, attitudes and clinical practice. *BMC International Health and Human Rights*.

[49] WHO (1979). *Seminar on Traditional Practices Affecting the Health of Women and Children*.

[50] OAU (June 2016). *The African Charter on the Rights and Welfare of the Child (ACRWC)*.

[51] UN (1994). *Report of the International Conference on Population*.

[52] UN (1995). *Report of the Fourth World Conference on Women*.

[53] Dugger C. W. (1996). *A Refugees Body Is Intact but Her Family Is Torn*.

[54] Ministry of Health Kenya (2010). *Management of Complications Pregnancy, Child-Birth and the Postpartum Period in the Presence of FGM/C*.

[55] National Council for Law Reporting (June 2016). *Prohibition of Female Genital Mutilation*.

[56] Kaplan Marcusan A., Riba Singla L., Laye M., Secka D. M., Utzet M., Le Charles M. A. (2016). Female genital mutilation/cutting: changes and trends in knowledge, attitudes, and practices among health care professionals in the Gambia. *International Journal of Women’s Health*.

[57] Lazar J. N., Johnson-Agbakwu C. E., Davis O. I., Shipp M. P.-L. (2013). Providers’ perceptions of challenges in obstetrical care for Somali women. *Obstetrics and Gynecology International*.

[58] Widmark C., Tishelman C., Ahlberg B. M. (2002). A study of Swedish midwives’ encounters with infibulated African women in Sweden. *Midwifery*.

[59] Zaidi N., Khalil A., Roberts C., Browne M. (2007). Knowledge of female genital mutilation among healthcare professionals. *Journal of Obstetrics and Gynaecology*.

[60] Isman E., Mahmoud Warsame A., Johansson A., Fried S., Berggren V. (2013). Midwives’ experiences in providing care and counselling to women with female genital mutilation (FGM) related problems. *Obstetrics and Gynecology International*.

[61] Hess R. F., Weinland J., Saalinger N. M. (2010). Knowledge of female genital cutting and experience with women who are circumcised: a survey of nurse-midwives in the united states. *Journal of Midwifery and Women’s Health*.

[62] WHO (November 2015). *Understanding and Addressing Violence Against Women: Female Genital Mutilation. 2012. WHO/RHR/12.35, 8*.

[63] Berg R. C., Underland V. (2014). *Immediate health consequences of female genital mutilation/cutting (FGM/C)*.

[64] Oduro A., Ansah P., Hodgson A. (2010). Trends in the prevalence of female genital muti-lation and its effect on delivery outcomes in the Kassena-Nankana district of northern Ghana. *Ghana Medical Journal*.

[65] Kaplan A., Hechavarria S., Bernal M., Bonhoure I. (2013). Knowledge, attitudes and practices of female genital mutilation/cutting among health care professionals in The Gambia: a multiethnic study. *BMC Public Health*.

[66] Leval A., Widmark C., Tishelman C., Maina A. B. (2004). The encounters that rupture the myth: contradictions in midwives’ descriptions and explanations of circumcised women immigrants’ sexuality. *Health Care for Women International*.

[67] Morrissey M. (2007). Suffer no more in silence: challenging the myths of women’s mental health in childbearing. *Illness by Psychiatry Nursing Staff*.

[68] Weissman M. M., Olfson M. (1995). Depression in women: implications for health care research. *Science*.

[69] Blackmore E. R., Rubinow D. R., O’Connor T. G. (2013). Reproductive outcomes and risk of subsequent illness in women diagnosed with postpartum psychosis. *Bipolar Disorders*.

[70] Berg R. C., Denison E. M.-L., Fretheim A. (2010). Psychological, social and sexual consequences of female genital mutilation/cutting (FGM/C): a systematic review of quantitative studies.

[71] Hopkins S. (1999). A discussion of the legal aspects of female genital mutilation. *Journal of Advanced Nursing*.

[72] Schwartz L. (2002). Is there an advocate in the house? The role of health care professionals in patient advocacy. *Journal of Medical Ethics*.

[73] Brown K., Beecham D., Barrett H. (2013). The Applicability of behaviour change in intervention programmes targeted at ending female genital mutilation in the EU: integrating social cognitive and community level approaches. *Obstetrics and Gynecology International*.

[74] Exterkate M. (June 2016). *Female Genital Mutilation in the Netherlands Prevalence, Incidence and Determinants, Pharos Centre of Expertise on Health for Migrants and Refugees*.

[75] Ith P., Dawson A., Homer C. S. (2012). Challenges to reaching MDG5: a qualitative analysis of the working environment of skilled birth attendants in Cambodia. *International Journal of Childbirth*.

[76] Onuh S. O., Igberase G. O., Umeora J. O., Okogbenin S. A., Otoide V. O., Gharoro E. P. (2006). Female genital mutilation: knowledge, attitude and practice among nurses. *Journal of the National Medical Association*.

